# Supplementary material for: Dynamic memristor-based reservoir computing for high-efficiency temporal signal processing
Source: Nat Commun. 2021 Jan 18;12:408. doi: 10.1038/s41467-020-20692-1 (PMC7814066; doi:10.1038/s41467-020-20692-1)
Supplement: Supplementary file 1 — Supplementary Information [file 41467_2020_20692_MOESM1_ESM.pdf]

# Supplementary Information

## Dynamic Memristor-based Reservoir Computing for High-Efficiency Temporal Signal Processing

Yanan Zhong[1], Jianshi Tang[1,2*], Xinyi Li[1], Bin Gao[1,2], He Qian[1,2], Huaqiang Wu[1,2*]

[1]Institute of Microelectronics, Beijing Innovation Center for Future Chips (ICFC), Tsinghua University, Beijing, 100084 China

[2]Beijing National Research Center for Information Science and Technology (BNRist), Tsinghua University, Beijing, 100084, China

[*]Email: jtang@tsinghua.edu.cn, wuhq@tsinghua.edu.cn.

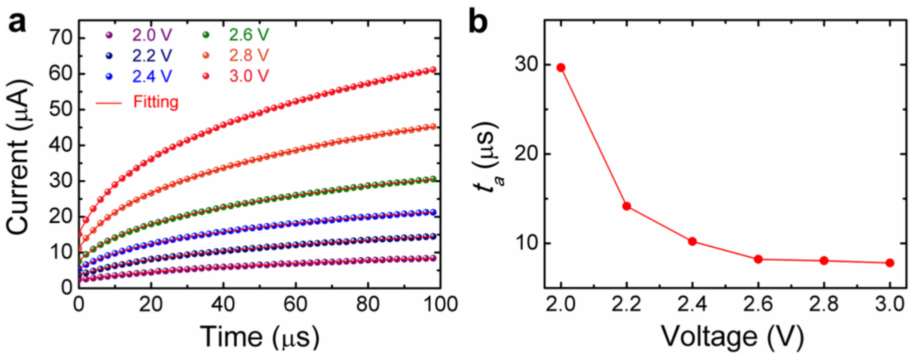


**Supplementary Figure 1**. **Integration behavior under different voltage pulse amplitudes.** (a) Current response under different voltage pulse amplitudes (2 V ~ 3 V). It can be seen that the increase of current shows two different regimes: when the voltage is just applied, the current shows a very fast increase and then the current increase becomes much slower after a few microseconds. Thus, the experimental results can be well fitted with a double exponential function shown as: $I = I_{\infty} - I_a \exp\left(-\frac{t}{t_a}\right) - I_b \exp\left(-\frac{t}{t_b}\right)$, where $t_a(t_b)$, $I_a(I_b)$, and $I_{\infty}$ represent the characteristic time, the prefactor, and a current constant for the fast-increase (slow-increase) process, respectively. (b) The characteristic time $t_a$, which determines the fastest pulse width we can apply on the device, decreases with the increasing applied voltage. When the applied voltage is greater than 2.4 V, $t_a$ is less than 10 μs. That means the pulse width (120 μs) of the input signal in our RC system can be further reduced to 10 μs or below, which would greatly reduce the system latency.

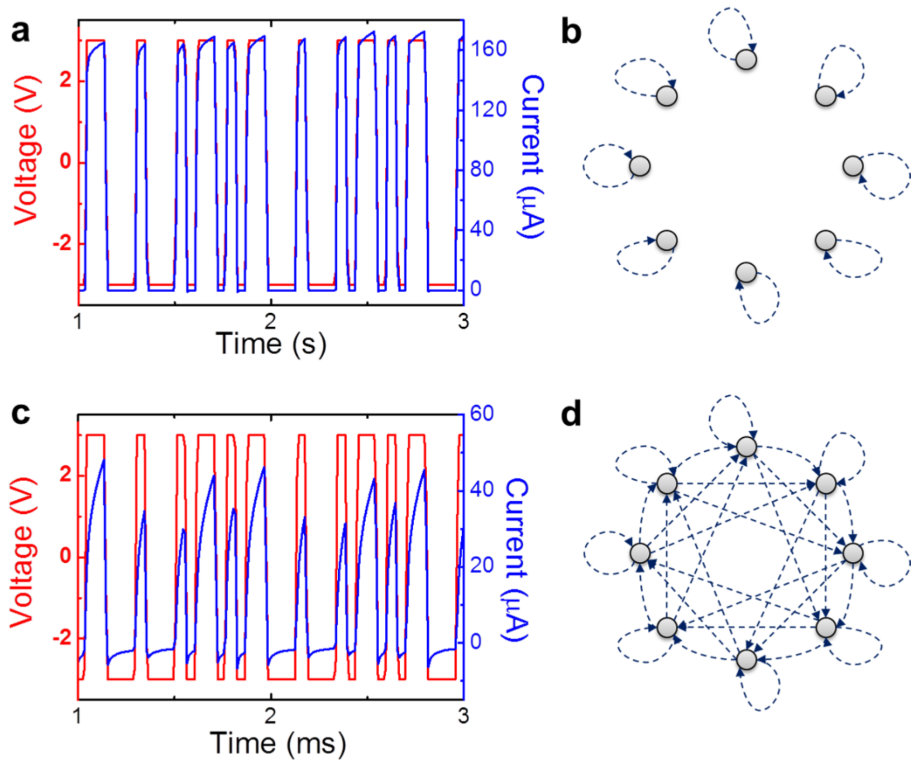


**Supplementary Figure 2**. **Investigation of the effect of the time step on the virtual nodes of the RC system.** (a) The input voltage after the mask process and the corresponding current response of the dynamic memristor, where the time step $\delta$ (50 ms) is much larger than the characteristic time $t_0$ (400 μs) of the device. In this case, the device rapidly saturates to a state that is independent of previous inputs. (b) As a result, the extracted virtual nodes are independent of each other, and each node is only coupled with itself at the previous time step. (c) The input voltage after the mask process and the corresponding current response of the dynamic memristor, where the time step $\delta$ (50 μs) is smaller than the characteristic time $t_0$ (400 μs) of the device. In this case, the device does not have enough time to reach a saturated state. (d) As a result, the extracted virtual nodes can be coupled with their neighbours efficiently, and hence a functional RC system can be implemented.

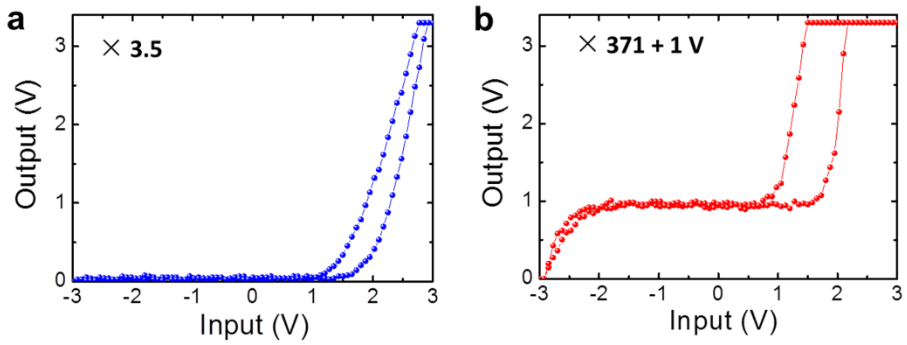


**Supplementary Figure 3**. **Nonlinear response region used for different tasks.** (a) The nonlinear response region used for waveform classification task. Through a load resistor, the output current of memristor is converted into a voltage signal which is then amplified by a factor of 3.5 times using an amplifier. In this way, the dynamic memristor response is mapped to a voltage range of 0 to 3.3 V for ADC sampling. (b) The nonlinear response region used for spoken-digit recognition and handwritten-digit recognition tasks. Here the memristor response is amplified by a factor of 371 while adding a voltage bias of 1 V. Because the values of input voltage mostly vary from -2 to 2V when performing these two tasks, we choose the appropriate amplifier gain and bias so that both positive and negative output responses can be well sampled by the ADC.

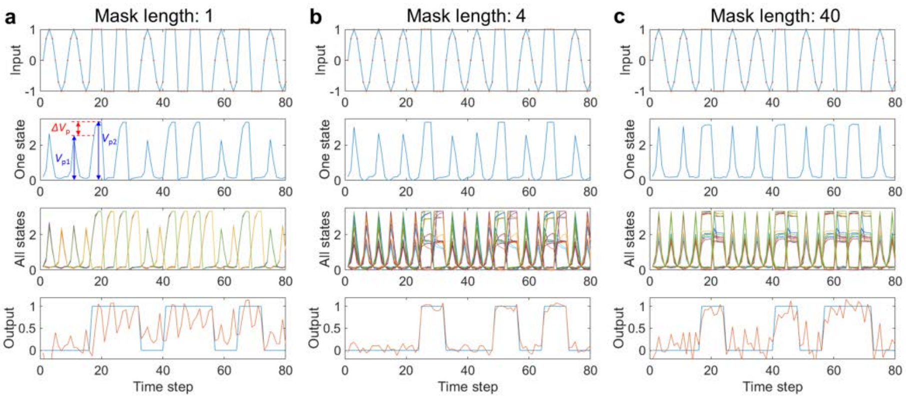


**Supplementary Figure 4**. **Waveform classification results under different mask length.** (a) - (c) Waveform classification results when the mask length changes from 1 to 40. The first to fourth panels of each figure show the input waveform, the first reservoir state, all reservoir states, and the classification results, respectively. As we can see from the second panel of each figure, one of the reservoir states extracted from the dynamic memristor response can transform the difference in waveforms into the change of amplitude ($\Delta V_p$). This effect can be described as the feedback strength between two duration $\tau$; however this feedback strength decreased with the increase of mask length. That is because the mask length can affect the overall change of memristor conductance over a duration $\tau$, as we mentioned in the main text. In addition, from the third panel of each figure we can find that more and more reservoir states overlap as the mask length decreases. When the mask length is 1, only two reservoir states can be distinguished, leading to a large error rate. This result further confirms our conclusion in the main text.

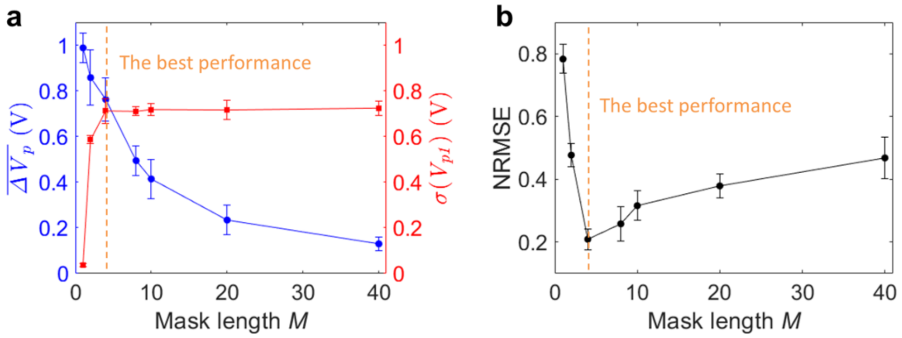


**Supplementary Figure 5**. **Quantitative analysis of feedback strength and state richness.** (a) The feedback strength and state richness changes with the mask length *M*, where the feedback strength and state richness are quantified as $\overline{\Delta V_p}$ and $\sigma(V_{p1})$ respectively. $\overline{\Delta V_p}$ is the average of the five largest $\Delta V_p$ in all reservoir states and $\sigma(V_{p1})$ is the standard deviation of $V_{p1}$ that is the peak response voltage of the sine wave. We can see that the feedback strength decrease with the mask length while the state richness increases with the mask length and saturates when the mask length is larger than 4. The best performance of the RC system is achieved when the mask length is around 4 that yields a trade-off between the feedback strength and the state richness. (b) The classification error (NRMSE) changes with the mask length, and NRMSE reaches the minimum value of 0.14 when the mask length is 4. The error bars in both (a) and (b) represent the variations between devices.

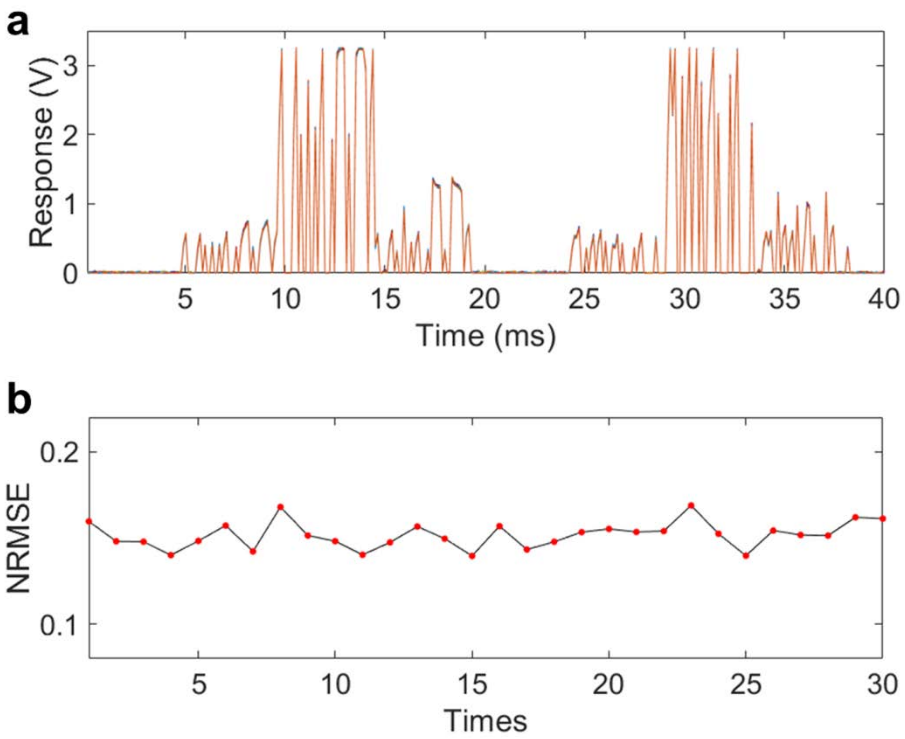


**Supplementary Figure 6**. **Cycle-to-cycle variation in waveform classification task.** (a) Measured memristor response from the waveform classification task, repeated by 30 times. (b) The variation of NRMSE during repeated tests.

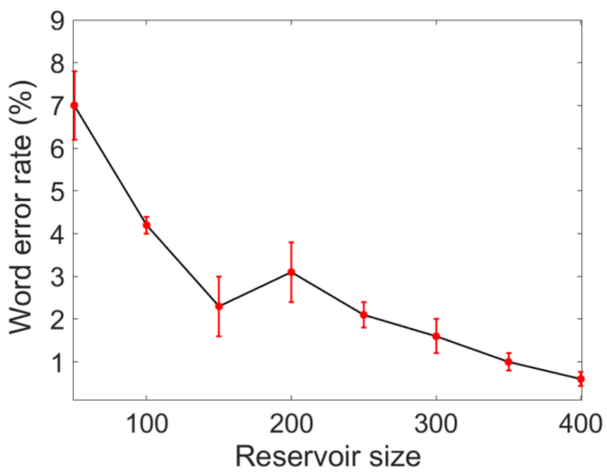


**Supplementary Figure 7**. **The tested word error rate changing with the reservoir size.** In each test, the mask length remains constant at 10, and the total reservoir size is adjusted by changing the number of parallel reservoirs *N*. The error bar shows the variation between devices.

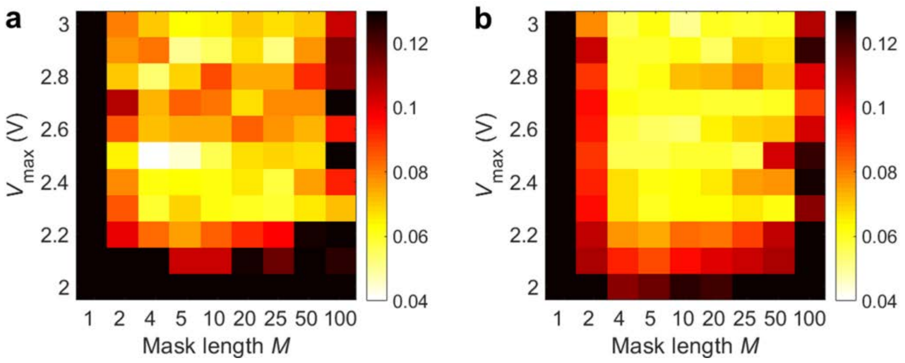


**Supplementary Figure 8**. **Device-to-device variation in Hénon map prediction task.** (a-b) Experimental results of the Hénon map prediction task obtained from two different devices, where the colour bar represents the NRMSE. $V_{max}$ and $M$ change from 2 to 3 V and 1 to 100 respectively. $V_{min}$ and $M \times N$ remain unchanged at -0.8 V and 100 respectively.

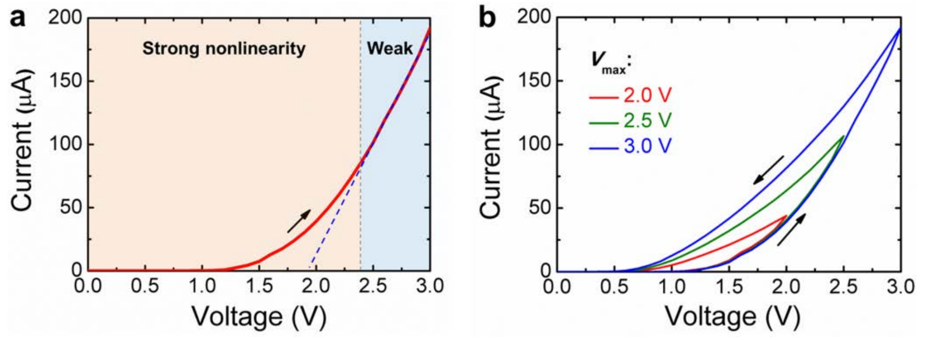


**Supplementary Figure 9**. **The *I*-*V* nonlinearity changing with voltage range.** (a) Typical *I*-*V* curve of the dynamic memristor during positive voltage sweep. The *I*-*V* curve can be roughly divided into two regions with strong nonlinearity and weak nonlinearity respectively. (b) The *I*-*V* loops under different voltage sweeping ranges $V_{max}$.

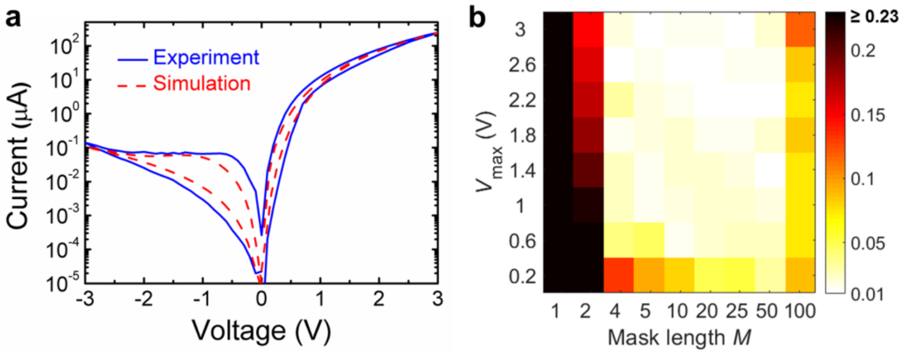


**Supplementary Figure 10. Simulated results of dynamic memristor-based RC system.** (a) The experimental and simulated I-V curves of the dynamic memristor. (b) The simulated result of the Hénon map prediction task, where the colour bar represents the NRMSE. $V_{\mathrm{max}}$ and $M$ change from 0.2 to 3 V and 1 to 100 respectively. $V_{\mathrm{min}}$ and $M \times N$ remain unchanged at 0 V and 100 respectively.

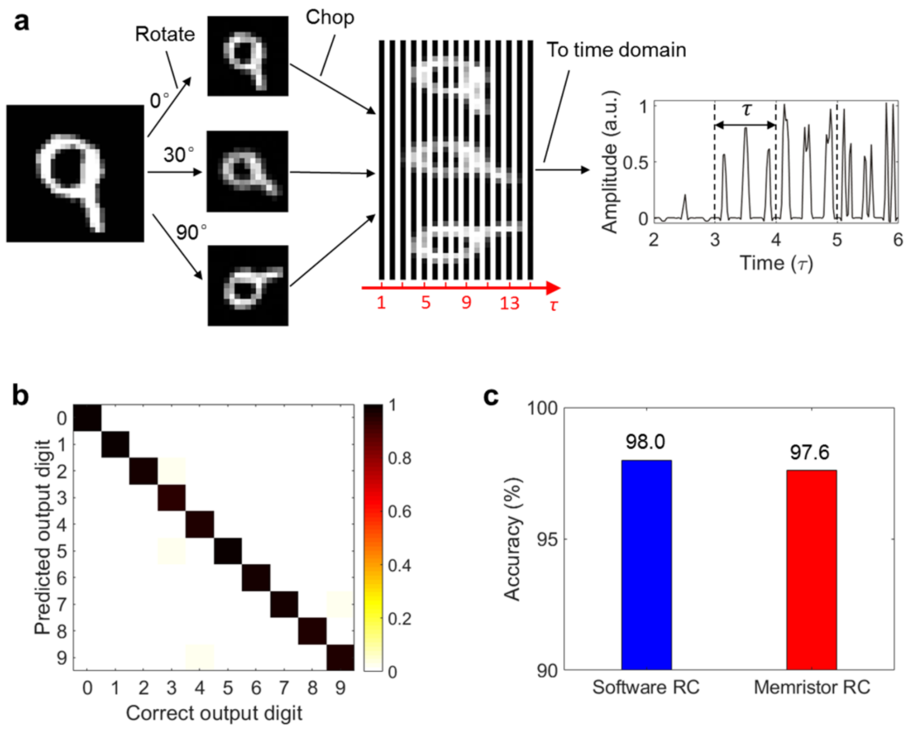


**Supplementary Figure 11. Handwritten-digit recognition demonstration.** (**a**) Schematic of converting the spatial pattern of a handwritten digit image to a temporal signal. Firstly, the original digit 9 is rotated by three different angles (0°, 30°, 90°).[1] Then, the three images are combined and chopped vertically. Finally, every piece of chopped image is transferred into a time-varying signal with a duration $\tau$ and the amplitude corresponds to the pixel grey-scale value. The mask process is similar as the one used in the previous spoken-digit recognition. During each time interval $\tau$, the pre-processed signal is multiplied by a $45 \times M$ mask matrix to generate the input voltage sequence with a time step $\delta$ ($\delta$ = 120 μs), where $M$ is equal to 4. $N$-parallel RC system is realized by using different mask matrices. Here $N$ is 300 to generate a total reservoir size of 1,200. The training and testing processes are similar to the spoken-digit recognition task and the only difference is that the feature vectors used for classification are composed of reservoir states selected within three time intervals

of $\tau$. (**b**) Predicted results obtained from the memristor-based RC system versus the correct outputs and the overall recognition accuracy reaches 97.6%. The colour bar represents the normalized probability of each predicted result under the correct output. (**c**) The comparison of the recognition accuracy between software-based and dynamic memristor-based RC systems, where the accuracy loss for our RC system is only 0.4% compared with standard software-based RC system.

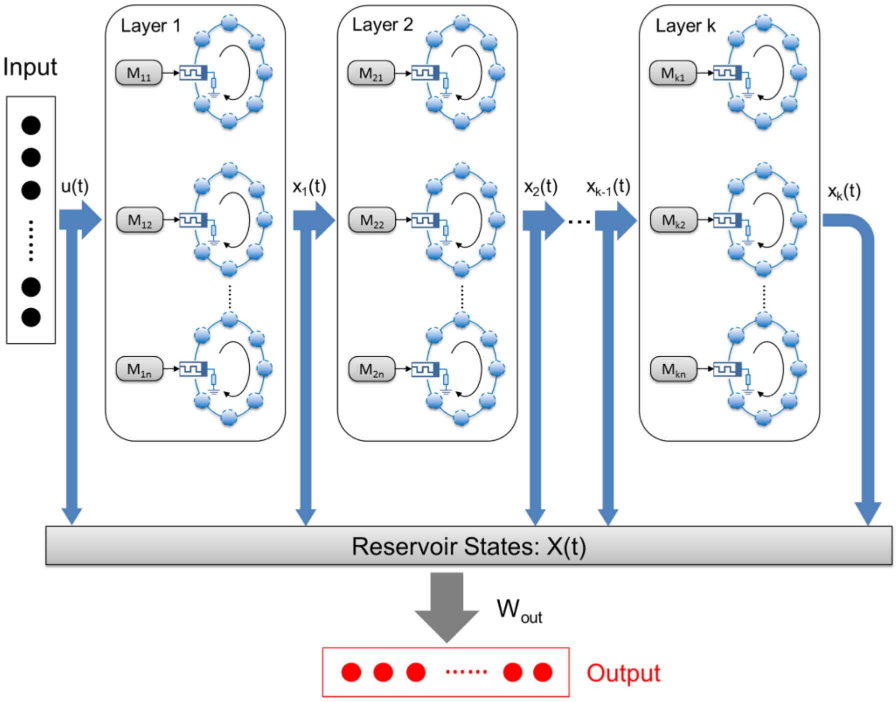


**Supplementary Figure 12. Schematic diagram of dynamic memristor-based multilayer RC system.** Each layer is composed of a memristor-based parallel RC system, and the difference between layers is determined by the mask matrix. The overall reservoir states consist of the original input and the state response of each layer. The system output is a linear combination of all reservoir states. Compared with the single-layer RC system, the multi-layer RC system would have an improved performance because of its richer reservoir states and stronger memory capacity.[2]

| **Parameters** | $G_0$ | $r$ | $\alpha$ | $K_p$ | $K_n$ |
|---|---|---|---|---|---|
| **Values** | 0.5 | 0.99 | 0.23 | 9.13 | 0.32 |

**Supplementary Table 1. The parameters used for simulation in Supplementary Figure 10.** The simple discrete model of dynamic memristor is defined as $I = KGV^3$ and $G = G_0 + r(G' - G_0) + \frac{\alpha|V|}{\alpha|V|+1}(G_{th} - G')$, where *I*, *V*, *G* and *G'* represent the output current, input voltage, the conductance at current time step and the conductance at pervious time step respectively. *K* and $G_{th}$ are the parameters varied with *V*. When *V* is positive, *K* and $G_{th}$ equal to $K_p$ and 1 respectively. When *V* is negative, *K* and $G_{th}$ equal to $K_n$ and 0 respectively.

| RC System | Power per input | Energy per input | Performance |
| --- | --- | --- | --- |
| Previous work[3] | 300 μW | 3 nJ | 99.2% |
| This work | 50 μW | 6 nJ | 99.6% |

**Supplementary Table 2. The estimated power and performance of different memristor-based RC systems in the spoken-digit recognition task.** In previous work, the power consumption for processing a single input is approximately 3.0 V × 100 μA = 300 μW, where the values of voltage and current are both taken from the reference. For the input pulse width in previous work is 10 μs, the energy consumption is then estimated as 300 μW × 10 μs = 3 nJ. In this work, because of the mask process, both the input voltage and the corresponding current vary within a certain range. Thus, the power consumption of processing a single input should be obtained by calculating the average power for all the inputs in a task. For the spoken-digit recognition task, the values of input voltage mostly vary from -2 to 2V (The corresponding current is in the range of -0.05 to 50 μA), so the average power is then estimated to be (-2 V × -0.05 μA + 2 V × 50 μA) ÷ 2 ≈ 50 μW for our RC system. The pulse width used in the current system is 120 μs (limited by measurement hardware), so the energy consumption per input pulse can be estimated as 50 μW × 120 μs = 6 nJ. Although the value is larger than that estimated in the previous work (300 μW × 10 μs = 3 nJ), there is still plenty of room for improvement in energy efficiency by reducing the input voltage pulse width (less than 10 μs) in future RC systems.

## Supplementary references


1. Schaetti N., Salomon M. & Couturier R., in *2016 IEEE Intl Conference on Computational Science and Engineering (CSE) and IEEE Intl Conference on Embedded and Ubiquitous Computing (EUC) and 15th Intl Symposium on Distributed Computing and Applications for Business Engineering (DCABES)*, 484-491 (IEEE, 2016).
2. Gallicchio C., Micheli A. & Pedrelli L. Design of deep echo state networks. *Neural Networks* **108**, 33-47 (2018).
3. Moon J. *et al.* Temporal data classification and forecasting using a memristor-based reservoir computing system. *Nat. Electron.* **2**, 480-487 (2019).
